# Paternal Resistance Training Induced Modifications in the Left Ventricle Proteome Independent of Offspring Diet

**DOI:** 10.1155/2020/5603580

**Published:** 2020-05-04

**Authors:** Ivo Vieira de Sousa Neto, Ramires Alsamir Tibana, Jonato Prestes, Leonardo Gomes de Oliveira da Silva, Jeeser Alves Almeida, Octavio Luiz Franco, Edilamar Menezes de Oliveira, Fabricio Azevedo Voltarelli, João Luiz Quaglioti Durigan, Marcelo Valle de Sousa, Carlos André O. Ricart, Katyelle Botelho, Mariana S. Castro, Wagner Fontes, Rita de Cassia Marqueti

**Affiliations:** ^1^Laboratory of Molecular Analysis, Graduate Program of Sciences and Technology of Health, Universidade de Brasília, Distrito Federal, Brazil; ^2^Graduate Program of Physical Education, Universidade Católica de Brasília, Distrito Federal, Brazil; ^3^Graduate Program in Health Sciences, Universidade Federal do Mato Grosso, Cuiabá, Brazil; ^4^Graduate Program in Health and Development in the Midwest Region, Faculty of Medicine, Universidade Federal do Mato Grosso do Sul, Campo Grande, Brazil; ^5^Research in Exercise and Nutrition in Health and Sports Performance-PENSARE, Graduate Program in Movement Sciences, Universidade Federal do Mato Grosso do Sul, Campo Grande, Brazil; ^6^Center for Proteomic and Biochemical Analyses, Graduate Program in Genomic Sciences and Biotechnology, Universidade Católica de Brasília, Distrito Federal, Brazil; ^7^S-Inova Biotech, Graduate Program in Biotechnology, Universidade Católica Dom Bosco, Campo Grande, Brazil; ^8^School of Physical Education and Sport, Universidade de São Paulo, São Paulo, Brazil; ^9^Laboratory of Protein Chemistry and Biochemistry, Department of Cell Biology, Institute of Biological Sciences, Universidade de Brasília, Distrito Federal, Brazil

## Abstract

Ancestral obesogenic exposure is able to trigger harmful effects in the offspring left ventricle (LV) which could lead to cardiovascular diseases. However, the impact of the father's lifestyle on the offspring LV is largely unexplored. The aim of this study was to investigate the effects of 8 weeks of paternal resistance training (RT) on the offspring left ventricle (LV) proteome exposed to control or high-fat (HF) diet. Wistar rats were randomly divided into two groups: sedentary fathers and trained fathers (8 weeks, 3 times per week with weights secured to the animals' tails). The offspring were obtained by mating with sedentary females. Upon weaning, male offspring were divided into 4 groups (5 animals per group): offspring from sedentary fathers, exposed to control diet (SFO-C); offspring from trained fathers, exposed to control diet (TFO-C); offspring from sedentary fathers, exposed to high-fat diet (SFO-HF); and offspring from trained fathers, exposed to high-fat diet (TFO-HF). The LC-MS/MS analysis revealed 537 regulated proteins among groups. Offspring exposure to HF diet caused reduction in the abundance levels of proteins related to cell component organization, metabolic processes, and transport. Proteins related to antioxidant activity, transport, and transcription regulation were increased in TFO-C and TFO-HF as compared with the SFO-C and SFO-HF groups. Paternal RT demonstrated to be an important intervention capable of inducing significant effects on the LV proteome regardless of offspring diet due to the increase of proteins involved into LV homeostasis maintenance. This study contributes to a better understanding of the molecular aspects involved in transgenerational inheritance.

## 1. Introduction

Obesity is a chronic disease characterized by excessive fat deposition in adipocytes, while an interplay between many systems is implicated in obesity etiology, including unbalanced energy uptake, and energy expenditure, gene mutations, aberrant gut microbiota, and epigenetic factors [1]. Overweight and obesity rates have grown dramatically in most western countries, reaching more than 2 billion people worldwide [2]. Obesity has been associated with several deleterious effects, including functional disability, reduction in life expectancy, and increased mortality [3, 4]. Therefore, there is an urgent need to understand its causes in order to develop preventive strategies.

There is an alarming trend of overweight in very young children, including infants [5]. Several new lines of research explain that obesity and metabolic syndrome can be programmed before the uterine life of an individual through transgenerational inheritance [6]. Evidence from animal and human studies suggests that environmental perturbations, such as diet, stress, and drugs before birth, contribute to the development of permanent metabolic consequences in offspring and result in negative effects on phenotype [7]. It is well established that paternal obesity is a risk factor for increased offspring adiposity, thereby predisposing to obesity [8]. The deleterious changes in metabolism are associated with glucose intolerance by dysfunction of pancreatic islets, with evident consequences in the first generation [9]. Offspring from obese fathers showed an increase of gonadal adiposity and the harmful effects of sperm metabolic function when compared to offspring from healthy fathers. These perturbed offspring phenotypes were associated with modifications of fathers' sperm microRNA content [10].

Exercise training is a remarkable nonpharmacological strategy to prevent and treat weight gain, as well as contribute to reducing cardiovascular disease risk, type 2 diabetes, cancers, and general health parameters. Exercise is an important piece in the obesity puzzle, representing a potent regulator of transgenerational inheritance for health and disease risk [10]. Krout et al. [11] reported in mice that paternal preconceptional exercise (2 weeks of wheel running) prevented the increase of type 2 diabetes risk in the offspring after paternal HF diet. This protection may occur by increases in the expression of insulin signaling pathways in the skeletal muscle of the offspring. Moreover, McPherson et al. [10] demonstrated that aerobic paternal exercise (8 weeks/swimming/3× week/30 min) exerted positive effects on serum cholesterol levels, body composition, and blood leptin concentration in offspring exposed to HF diet.

To note, ancestral obesogenic exposure is also able to trigger the onset and/or induce important changes in the offspring left ventricle (LV) that could lead to cardiovascular diseases [12]. In rodent models of ancestral obesity, descendants showed an increased risk of myocardial dysfunction, such as LV hypertrophy and myocardial fibrosis [13]. Maternal HF diet exposure resulted in decreased cardiac function and compromised mitochondrial integrity in the LV, accompanied by increased lipid content in the next generation [14]. It has been demonstrated that children from obese females are at a greater risk for adverse cardiovascular outcomes and congenital heart defects [15] accompanied by increased risk of premature death [16]. However, the impact of the father's lifestyle on offspring LV adaptations remains poorly understood.

Although multiple studies determined that chronic exercise can be a crucial factor in transgenerational inheritance, the molecular mechanisms underlying cardiovascular benefits generated from exercise training remain to be investigated. In this sense, proteomics is an efficient technique with satisfactory precision that estimates a large number of proteins at the same time and represents an import method to clarify more profoundly the molecular networks behind physiological adaptations promoted by exercise training [17]. In a recent study, UPLC-MSE proteomics revealed modulation on the LV proteome from rats submitted to resistance training (RT) [18]. The authors demonstrated that RT upregulates protein abundance levels related to metabolic processes, myofibril components, transporter, and antioxidant activity on the LV, which indicates that RT exerts important cardiac protective effects [18].

To date, we found no investigation regarding the effects of a paternal resistance training program in the offspring proteomic profile. Furthermore, there is no proteomic study on the LV in which offspring were exposed to HF diet. This information would be valuable to elucidate potential mechanisms induced by paternal training that could attenuate harmful alterations inherent to offspring high-fat diet. The global aim of this study was to investigate the effects of 8 weeks of paternal RT preconception on the offspring LV proteome exposed to control and HF diets. We hypothesize that paternal RT upregulates protein abundance levels associated with myofibril components, metabolic processes, antioxidant activity, transport, nucleosome assembly, translation, and transcription in the offspring. Moreover, we expect to find differences in offspring protein abundance levels exposed to different diets.

## 2. Materials and Methods

### 2.1. Animals and Grouping

All procedures were conducted in accordance with the USA Guide for Care and Use of Laboratory Animals [19]. The research protocol received approval from the Ethics Committee on Animal Experimentation from a local university (protocol no. 010/13). Initially, 4-month-old male Wistar rats (*Rattus norvegicus albinus*, weighing ±376 g) were placed in collective cages (maximal 4 rats per cage), and were randomly divided into two groups (5 animals per group): sedentary fathers (SF; did not perform RT) and trained fathers (TF; performed RT). The offspring were obtained by mating with sedentary females. After the 8 weeks of paternal RT, the estrous cycle of females was checked daily, and during the proestrus phase, one male and one female were housed together for two consecutive days, during which they were allowed free access to a control diet (Purina®, Descalvado-SP, Brazil) and water. The experimental groups in this study were composed of 20 male pups. Litters were standardized among 5 pups each to avoid litters of disparate sizes, which were left together with their mothers until they were weaned at the age of twenty-one days. Litters belonging to the same experimental group were offspring of different parents.

**Figure 1:**
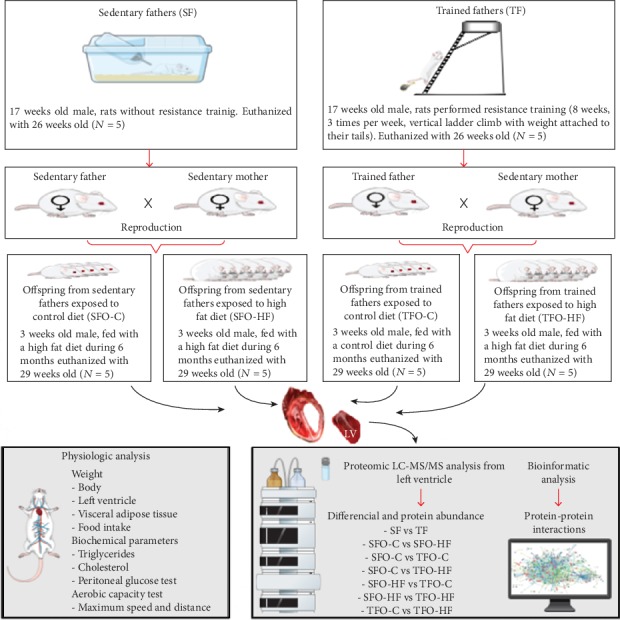
Experimental design. Schematic illustration of the methodological steps in the following study.

Upon weaning, male offspring were divided into 4 groups (5 animals per group): offspring from sedentary fathers exposed to control diet (SFO-C), offspring from trained fathers exposed to control diet (TFO-C), offspring from sedentary fathers exposed to high-fat diet (SFO-HF), and offspring from trained fathers exposed to high-fat diet (TFO-HF). All animals came from the Central Vivarium of the Faculty of Physical Education of the Catholic University of Brasilia. The animals were housed in polypropylene cages at a temperature of 23 ± 2°C with a 12 : 12 h dark : light cycle. The offspring were weighed in grams and evaluated weekly for 6 months using a digital scale (Filizola®, São Paulo, Brazil). Study experimental design is showed in Figure 1.

### 2.2. Offspring Diet

The offspring exposed to control diets (66.00% carbohydrates, 24.00% protein, and 10.00% lipids, totaling 3.48 kcal/g) were fed with standard feed (Labina Presence®, Paulinia, São Paulo, Brazil) and water ad libitum. The SFO-HF and TFO-HF groups were exposed to HF diets commercially purchased (20.27% carbohydrates, 19.89% protein, and 59.38% lipids, totaling 5.20 kcal/g) (Prag solutions®, Biosciences, Jau, Brazil), overload of 200 ml of soft drink per week (100% carbohydrates 21 g, sodium 10 mg, totaling 0.85 kcal/g), and water ad libitum after the 21st day of birth, during 6 months. Compositions of the experimental diets are compiled in Supplementary data 1. Previous studies demonstrated the effectiveness of this HF diet on body weight gain, adipose tissue weight gain, and plasma lipids in Wistar rats [20, 21]. Females were also kept on a control diet (Labina Presence®, Paulinia, São Paulo, Brazil) throughout gestation and lactation. The offspring foods were weighed weekly in grams, using a digital scale (Filizola®, São Paulo, Brazil), and food intake (amount offered–amount remaining in the cage) was monitored.

### 2.3. Paternal Resistance Training Protocol

The exercise protocol was designed according to Hornberger and Farrar [22]. Training procedures were also described elsewhere [23–25]. During the 8 weeks, climbing sessions were performed 3 times per week. Prior to the training period, a RT adaptation protocol was administered, which required the animals to climb a vertical ladder (1.1 m × 0.18 m, 2 cm grid, 80° incline) with loads attached to their tails. The size of the ladder required the animals to perform 8–12 movements. The load apparatus was secured to the tail by wrapping the proximal portion of the tail with a self-adhesive foam strip. If necessary, a stimulus was applied, with tweezers, to the animal's tail to initiate the movement. At the top of the ladder, the rats reached a housing chamber, where they were allowed to rest for 120 s. The rats performed 3 sections with a 48 h interval. The first RT session started 3 days after the familiarization.

The initial climb consisted of a load that was 75% of the animal's body mass. After this, an additional 30 g weight was added until the rat could not climb the entire ladder with such load. Failure was determined when the animal could not progress up the ladder after 3 successive stimuli to the tail. The highest load successfully carried was considered to be the rat's maximal carrying capacity. After the maximum load capacity, training sessions for fathers consisted of 8 ladder climbs with 2 sets of each load 50, 75, 90, and 100% of their maximal carrying capacity interspersed by 120 s of intervals between each set.

### 2.4. Offspring Aerobic Capacity

Incremental-speed treadmill running was used for assessing the maximal aerobic capacity in offspring and was completed in the last two weeks of diet exposure. The test was adapted from Almeida et al. [26]. In order to minimize stress related to physical exercise during the test, an adaptation protocol was adopted. Before the test, the animals were initially familiarized with running on a treadmill designed for small animals (Li 870, Letica Scientific, Barcelona, Espanha) during 3 days with a constant speed of 13 m·min^−1^ during 10 min. The test session started 2 days after the familiarization period. During the incremental exercise, the rats started running at a speed of 13 m·min^−1^, followed by speed increments of 3 m·min^−1^ every 3 min until they reached fatigue. Volitional fatigue was defined as the point at which the animals were no longer able to maintain their pace with the treadmill, even when exposed to light electrical stimulation for 10 s. The maximal aerobic capacity was established by the antecedent stage.

### 2.5. Offspring Blood Sample Collection and Biochemical Analysis

At the end of the 24-week exposure to diet, offspring were fasted overnight for the evaluation of blood glucose and lipid profile. The intraperitoneal glucose tolerance test was performed via a small incision on the distal end of the animal's tail. Animals received an injection of 20% D-glucose (2 g/kg body weight), and approximately 5 *μ*l of tail blood was collected to measure the blood glucose concentrations at 15, 30, 60, and 120 min following glucose injection. Blood glucose, triglycerides, and cholesterol were measured using a glucometer (ACCU-CHECK Active, Roche®, Mannheim, Germany), and their respective reagent tapes, according to the manufacturer's recommendations.

### 2.6. Euthanasia

To avoid the acute effects of RT, the fathers were euthanized with an intraperitoneal injection of xylazine solution (12 mg/kg of body weight) and ketamine (95 mg/kg of body weight) 48 h after the end of the training period. The offspring were euthanized using the same combination of solutions after 24 weeks of exposure to diet. The LV and visceral adipose tissue of the offspring were dissected and immediately washed with saline. The samples were weighed and frozen in liquid nitrogen and stored in a freezer at −84°C.

### 2.7. Left Ventricle Protein Extraction

Proteomic analysis and bioinformatics tools were adapted from Cury et al. [27] according to the needs of our research. Approximately 100 mg of the LV from fathers and offspring was macerated in liquid nitrogen and mechanically homogenized using a mortar and pestle. Posteriorly, the sample was added to a lysis solution containing 10% (*w*/*v*) trichloroacetic acid and 0.07% (*v*/*v*) *β*-mercaptoethanol in cold acetone; the resulting suspension was thoroughly mixed by vortexing and incubated for 3 h at 4°C. In the next incubation, samples were centrifuged (10,000 g for 20 min at 4°C). The supernatant was removed, and the pellet was washed five times with 10% (*w*/*v*) trichloroacetic acid in acetone until the disappearance of pigments. The pellet was dried using a concentrator and resuspended in rehydration solution (7 M urea, 2 M thiourea, 250 mM TEAB, pH 8.5). The protein concentration from each extraction was assayed using Qubit® 2.0 (Invitrogen, Carlsbad, USA). The purity of protein extracted was assessed in gel 10% SDS-PAGE.

### 2.8. Protein Digestion

The extracted proteins from the LV (200 *μ*g) were prepared for proteomic analysis, as described in Cury et al. [27]. The sample was quantified by Qubit® 2.0 (Invitrogen, Carlsbad, USA) for analysis by nanoscale liquid chromatography coupled to mass spectrometry.

### 2.9. Nano-LC-MS/MS Analysis

The chromatography and mass spectrometry analysis was adapted from Curry et al. [27] The tryptic peptides were applied to Dionex UltiMate 3000 Liquid Chromatographer (Sunnyvale, USA) for reversed phase nanochromatography. One microgram from the sample was injected into a column (2 cm × 100 *μ*m, containing C18 5 *μ*m particles), connected to the analytical column (32 cm × 75 *μ*m, C18 3 *μ*m), and eluted to the ionization source of the spectrometer. The elution solution consisted of 0.1% (*v*/*v*) formic acid in water (solvent A) and 0.1% (*v*/*v*) formic acid in acetonitrile (solvent B), in a gradient of 2% to 35% solvent B for 180 min.

The eluted fractions were sprayed in the ionization source of the LTQ Orbitrap Elite mass spectrometer (Thermo Fisher Scientific, Germany). Mass spectrometry analysis was performed in data-dependent acquisition mode, when peptides were applied to the Orbitrap analyzer in the range of 300-1650 *m*/*z* with a resolution of 120,000 FWHM. The fifteen more intense ions were automatically submitted to high-energy collision-induced dissociation fragmentation through an insulation window of 2.0 *m*/*z*, gain control of 5 × 10^6^, normalized collision energy of 35%, and threshold for the selection of 3000.

### 2.10. Database Search and Label-Free Quantification

The files of the mass spectrometer were analyzed by software Progenesis QI [28] for alignment of the MS1 peaks found in the chromatograms. The peptide peaks were quantified and grouped. Protein identification was achieved using Peaks 7.0 software [29], to perform sequencing and PSM database search from the fragmentation information. The database was downloaded from UniProt, filtered by *Rattus* spp. taxonomy. Precursor ion mass error tolerance of 10 ppm, MS/MS mass tolerance of 0.05 Da, carbamidomethylation of cysteine residues (fixed modification), and deamidation and methionine oxidation (variable modifications) were used as search parameters. The peak area of each MS1 ion was calculated, and these values were used for the intensity calculations. The identified proteins were filtered at 1% for false discovery rate, and a minimum of one peptide per protein was required for identification. The inclusion criteria for identified proteins were the presence in at least four of five animals from each group. The proteins were grouped in biological process class according to Petriz et al. [17].

### 2.11. Protein Interaction Analysis

Protein interaction analysis was adapted from Cury et al. [27] according to the requirements of our study. Upregulated proteins were investigated using bioinformatics tools such as STRING 10.0 (Search Tool for the Retrieval of Interacting Genes/Proteins) [30]. Protein–protein interactions were performed using the medium confidence score (0.400) filtered with Rattus norvegicus database.

### 2.12. Statistical Analysis

The results from proteomic analysis were presented according Petriz et al. [17]. Kolmogorov-Smirnov and Levene tests were used to analyze the homogeneity of the variance. A two-way mixed ANOVA was used to compare body weight evaluated weekly for six months and serum glucose levels in the intraperitoneal glucose tolerance test. The Mauchly test verified compound sphericity. When the assumption of sphericity was not met, the significance of *F* ratios was adjusted according to the Greenhouse-Geisser procedure. Independent *t*-test was used for comparison of protein abundance levels between father groups. A two-way independent ANOVA (training vs. diet as factors) was used to compare food intake, tissue weight, triglycerides, cholesterol, aerobic capacity, and protein abundance levels between offspring groups. Tukey post hoc test was applied to identify the differences. Two-way ANCOVA was applied to determine whether there was an interaction effect between offspring diet and paternal exercise on protein abundance levels while controlling for covariates (body weight and tissue weight). Simple main effects were performed with Bonferroni adjustment. The proteins with a fold change of at least 2 in the log(*e*) ratio between the treatments were considered upregulated and downregulated. An alpha threshold of 0.05 was considered for significance. The Statistical Package for the Social Sciences (SPSS Inc., v. 21.0; IBM Corporation, Armonk, NY, USA) was used for statistical analysis, and GraphPad Prism 6.0 (San Diego, CA, USA) was used for graphic design.

## 3. Results

### 3.1. Body and Tissue Weights of Offspring Groups

**Figure 2:**
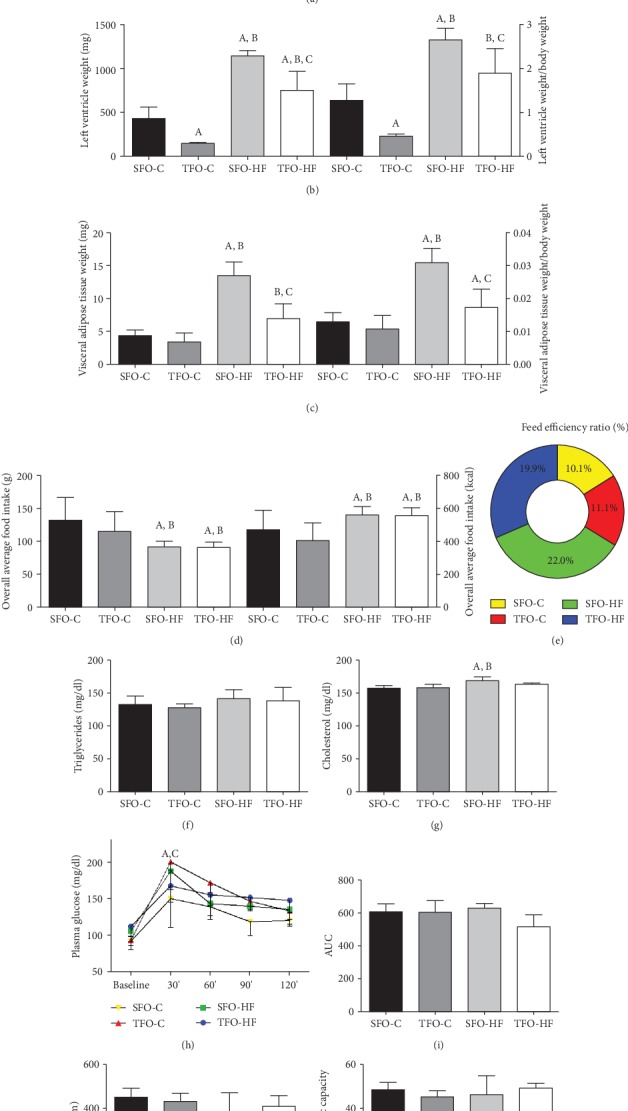
Physiologic analysis in offspring exposed to control and high-fat diets. Body weight (a), left ventricle weight (b), visceral adipose tissue weight (c), overall average food intake in grams and kcal (d), feed efficiency ratio (e), triglycerides (f), cholesterol (g), plasma glucose (h), the total area under the curve in the intraperitoneal glucose tolerance test (h), distance (j), and maximum speed in the aerobic capacity test (k). Values are presented as mean ± SD. SFO-C: offspring from sedentary fathers, exposed to control diet; TFO-C: offspring from trained fathers exposed to control diet; SFO-HF: offspring from sedentary fathers exposed to high-fat diet; TFO-HF: offspring from trained fathers exposed to high-fat diet. Statistically significant differences compared to ^a^SFO-C, ^b^TFO-C, and ^c^SFO-HF. *p* ≤ 0.05 (*N* = 5 per group).

There was a significant interaction between intervention groups and weeks on body weight for male offspring. The main effects showed that RT prevented body weight gain in the TFO-HF (398.8 ± 8.1) group as compared with the SFO-HF group (433.9 ± 34.7) after 24 weeks of high-fat diet exposure (*p* = 0.001). There was no difference between the TFO-C and SFO-C groups on body weight (Figure 2(a)).

Offspring exposed to HF diet displayed higher left ventricle weight as compared with the offspring exposed to control diet (*p* = 0.001). Furthermore, offspring left ventricle weight was modified by paternal training, as shown by decreased left ventricle weight in the TFO-C and TFO-HF groups as compared with the SFO-C and SFO-HF groups (*p* = 0.02 and *p* = 0.001, respectively). Also, the SFO-HF group displayed higher left ventricle weight when compared with the TFO-C group (*p* = 0.001) (Figure 2(b)).

There was a significant interaction between paternal training and offspring diet for visceral adipose tissue weight. The SFO-HF group showed increased visceral adipose tissue when compared with the SFO-C and TFO-C groups (*p* = 0.001 and *p* = 0.001, respectively). Moreover, the TFO-HF group demonstrated increased visceral adipose tissue weight compared with the TFO-C group (*p* = 0.020) and lower visceral adipose tissue as compared with the SFO-HF group (*p* = 0.001) (Figure 2(c)).

### 3.2. Overall Food Intake and Feed Efficiency Ratio (%)

Offspring exposed to the HF diet consumed fewer grams of food overall when compared with offspring exposed to the control diet (*p* = 0.001). However, animals consuming HF diet had an increase in overall caloric consumption (*p* = 0.001). There were no differences in food intake between offspring from sedentary fathers and offspring from trained fathers in both diets (*p* > 0.05) (Figure 2(d)). Offspring exposed to the HF diet showed a higher feed efficiency ratio when compared with animals exposed to the control diet (22% vs. 10%) (Figure 2(e)). Regarding soft drink intake, the SFO-HF and TFO-HF groups consumed 200 ml offered per week.

### 3.3. Biochemical Parameters

There was no difference between groups on serum triglycerides (Figure 2(f)). Serum cholesterol levels increased significantly in the SFO-HF group as compared with the SFO-C and TFO-C groups (*p* = 0.001 and *p* = 0.01, respectively); however, there was no difference between the SFO-HF and TFO-HF groups (*p* > 0.05) (Figure 2(g)).

The TFO-C group displayed higher levels of serum glucose after 30 min when compared with the SFO-C group (*p* = 0.001). Moreover, the SFO-HF group showed increased levels of serum glucose after 30 min when compared with the SFO-C group (*p* = 0.001) (Figure 2(h)). There were no differences in the area under the curve (AUC) of glucose response between groups (*p* > 0.05) (Figure 2(i)).

### 3.4. Offspring Aerobic Capacity

There was no change in maximal distance and aerobic capacity in the experimental groups (*p* > 0.05) (Figures 2(j) and 2(k)).

### 3.5. Functional Proteome Description

Data reported here revealed 557 proteins identified by LC-MS/MS analysis (Supplementary data 2); however, 536 proteins met the inclusion criteria and were classified according to the UniProt database and Panther classification system in biologic process, molecular function, and cellular localization. Supplementary data 3A shows the identified proteins according to their cellular localization (A), biologic process (B), and molecular function (C). In the present investigation, the majority of these proteins were derived from mitochondria (30.04%) followed by cytoplasm (20%), nucleus (10.43%), and cytoskeleton (10.07%) (Supplementary figure 1A). Additionally, the biological process classification indicated that these proteins were related to metabolic processes (24.91%), followed by transport (11.62%), biological regulation (9.60%), cell component organization (8.30%), and others (7.01%) (Supplementary figure 1B). Finally, the main molecular function activity observed was enzymatic (35%), followed by binding (29.81%), structural (7.04%), transporter activity (6.49%), and chaperone activity (5.18%) (Supplementary figure 1C).

Venn diagram was used to show the distribution of the identified proteins into two experimental groups (Supplementary figure 2). From the 536 identified proteins, 531 were common to all groups, while 4 proteins were identified only in the sedentary father (SF) group and 1 protein was identified only in the trained father (TF) group. The identified proteins only in the SF group were mainly related to the apoptotic process (apoptosis-inducing factor 1), cell shape (mitochondrial erythrocyte membrane protein band 4.1), oxidation-reduction process (NADH dehydrogenase (ubiquinone) 1 alpha subcomplex subunit 5), and metabolic process (transketolase). The protein identified only in the TF group was associated with transport (kinesin-like protein).

Regarding the four offspring groups, from the 536 identified proteins, 532 were common to all groups, with 1 protein identified only in the offspring from trained fathers exposed to high fat-diet (TFO-HF) group. The identified protein exclusive to the TFO-HF group was related to metabolic process (NADH dehydrogenase (ubiquinone) 1 alpha subcomplex subunit 5) (Supplementary figure 3).

### 3.6. Paternal Left Ventricle Proteome

**Figure 3:**
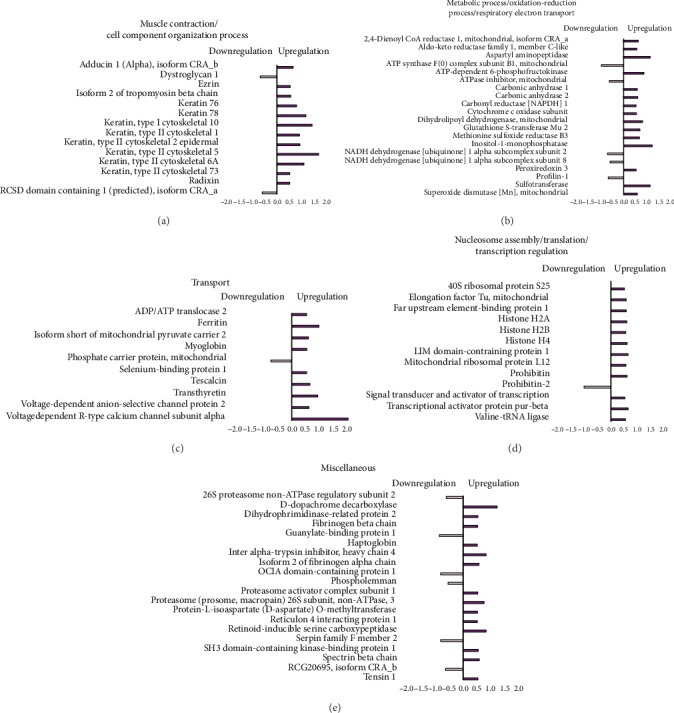
Effects of resistance training on the LV proteome. Histogram of protein abundance levels from intergroup analysis considering only proteins with downregulation (light purple) and upregulation (purple) (*p* ≤ 0.05), with a delta (Δ) of at least (≥) 0.5-fold change. SF: sedentary fathers; TF: trained fathers. The *x*-axis represents the log(*e*) ratio between the treatments (TF : SF ratio). All altered proteins are grouped according to their biologic process as noted in Gene Ontology (GO). Muscle contraction and cell component organization; metabolic process, oxidation-reduction process, and respiratory electron transport; transport; nucleosome assembly, translation, and transcription regulation; and miscellaneous.

The effects of resistance training on paternal LV protein abundance levels were grouped according to their biological processes and are presented in Figure 3. From the TF group, abundance levels of 77 proteins were shown to be altered as compared with the SF group (62 proteins increased and 15 decreased). In this analysis (TF : SF), proteins were mainly related to muscle contraction and cell component organization (12 upregulated and 2 downregulated); metabolic process, respiratory electron transport, and oxidation-reduction process (15 upregulated and 5 downregulated); transport (9 upregulated and 1 downregulated); nucleosome assembly, translation, and transcription regulation (12 upregulated and 1 downregulated); and miscellaneous (14 upregulated and 6 downregulated).

### 3.7. High-Fat Diet Caused Disturbance of Proteins Related to Transport, Translation, and Miscellaneous in the Offspring

**Figure 4:**
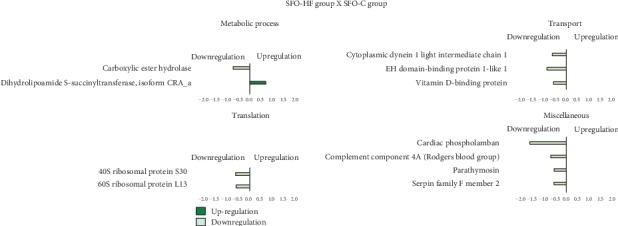
Effects of high-fat diet on the LV proteome in the offspring. Histogram of protein abundance levels from intergroup analysis considering only proteins with downregulation (light green) and upregulation (green) (*p* ≤ 0.05), with a delta (Δ) of at least (≥) 0.5-fold change. SFO-C: offspring from sedentary fathers exposed to control diet; SFO-HF: offspring from sedentary fathers exposed to high-fat diet. The *x*-axis represents the log(*e*) ratio between the treatments (SFO-HF : SFO-C ratio). All altered proteins are grouped according to their biologic process as noted in Gene Ontology (GO). Metabolic process, transport, translation, and miscellaneous.

High-fat diet modifies an abundance of 10 proteins (1 protein increased and 9 decreased). In this analysis (SFO-HF : SFO-C), the proteins were mainly related to metabolic process, respiratory electron transport, and oxidation-reduction process (1 upregulated), transport (3 downregulated), translation (2 downregulated), and miscellaneous (4 downregulated) (Figure 4).

### 3.8. Paternal RT Modulates Numerous Biological Pathways in the Left Ventricle Proteome of Offspring Exposed to Control Diet

**Figure 5:**
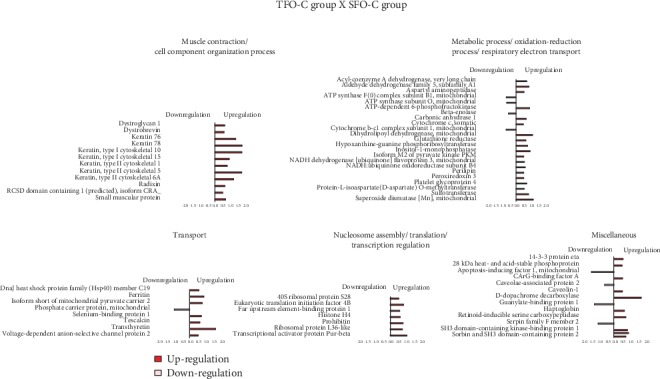
Effects of paternal resistance training on the LV proteome in the offspring exposed to control diet. Histogram of protein abundance levels from intergroup analysis considering only proteins with downregulation (light red) and upregulation (red) (*p* ≤ 0.05), with a delta (Δ) of at least (≥) 0.5-fold change. SFO-C: offspring from sedentary fathers exposed to control diet; TFO-C: offspring from trained fathers exposed to control diet. The *x*-axis represents the log(*e*) ratio between the treatments (TFO-C : SFO-C ratio). All altered proteins are grouped according to their biologic process as noted in Gene Ontology (GO). Muscle contraction and cell component organization; metabolic process, oxidation-reduction process, and respiratory electron transport; transport; nucleosome assembly, translation, and transcription regulation; and miscellaneous.

Paternal RT upregulates protein abundance related to myofibril components, antioxidant activity, transport, and transcription even when puppies are exposed to the standard diet. From the TFO-C group, abundance levels of 57 proteins were shown to be altered when compared with the SFO-C group (50 proteins increased and 7 decreased). In this analysis (TFO-C : SFO-C), proteins were mainly related to muscle contraction and cell component organization (12 upregulated); metabolic process, respiratory electron transport, and oxidation-reduction process (17 upregulated and 3 downregulated); transport (7 upregulated and 1 downregulated); nucleosome assembly, translation, and transcription regulation (6 upregulated); and miscellaneous (8 upregulated and 3 downregulated) (Figure 5).

### 3.9. Paternal RT Promoted the Positive Regulation of Essential Proteins Associated to Muscle Contraction, Antioxidant Activity, Transport, and Transcription in the Offspring Exposed to a High-Fat Diet

Most of the regulated proteins in TFO-HF presented an increased abundance rather than reduced when compared to other experimental groups. Most of the modulated proteins are associated with biological pathways related to the heart protection process.

**Figure 6:**
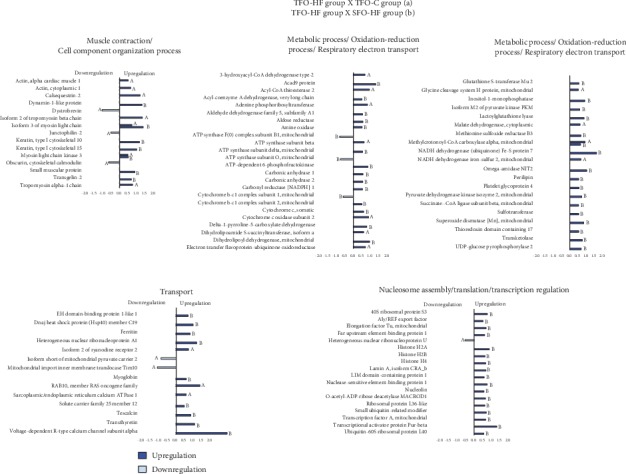
Effects of paternal resistance training on the LV proteome in the offspring exposed to high-fat diet. Histogram of protein abundance levels from intergroup analysis considering only proteins with downregulation (light blue) and upregulation (blue) (*p* ≤ 0.05), with a delta (Δ) of at least (≥) 0.5-fold change. TFO-C: offspring from trained fathers exposed to control diet; SFO-HF: offspring from sedentary fathers exposed to high-fat diet; TFO-HF: offspring from trained fathers exposed to high-fat diet. The *x*-axis represents the log(*e*) ratio between the treatments (a, TFO-HF : TFO-C ratio; b, TFO-HF : SFO-HF ratio). All altered proteins are grouped according to their biologic process as noted in Gene Ontology (GO). Muscle contraction and cell component organization; metabolic process, oxidation-reduction process, and respiratory electron transport; transport; and nucleosome assembly, translation, and transcription regulation.

The TFO-HF group displayed modified abundance levels of 70 proteins when compared with the SFO-HF group (67 proteins increased and 3 decreased) (Figure 6, Table 1). Furthermore, in the TFO-HF group, abundance levels of 36 proteins were shown to be altered when compared with the TFO-C group (31 proteins increased and 5 decreased), respectively. When the TFO-HF group was compared with the SFO-HF group, proteins were mainly related to muscle contraction and cell component organization (5 upregulated); metabolic process, respiratory electron transport, and oxidation-reduction process (23 upregulated and 3 downregulated); transport (8 upregulated); nucleosome assembly, translation, and transcription regulation (16 upregulated); and miscellaneous (15 upregulated) (Table 1). There was statistically significant interaction between paternal training and offspring diet on 43 proteins while controlling for body and tissue weights (Table 2). Moreover, when the TFO-HF group was compared with the TFO-C group, proteins were mainly related to muscle contraction and cell component organization (6 upregulated and 2 downregulated); respiratory electron transport and oxidation-reduction process (8 upregulated); transport (3 upregulated and 2 downregulated); nucleosome assembly, translation, and transcription regulation (1 downregulated); and miscellaneous (14 upregulated and 1 downregulated). There was a statistically significant interaction between paternal training and offspring diet on 43 proteins while controlling for body and tissue weights (Table 2). Values of the mean square, *F*, significance, partial eta squared, and observed power of two-way ANCOVA analyses are reported in Supplementary data 3.

### 3.10. Protein-Protein Interactions

**Figure 7:**
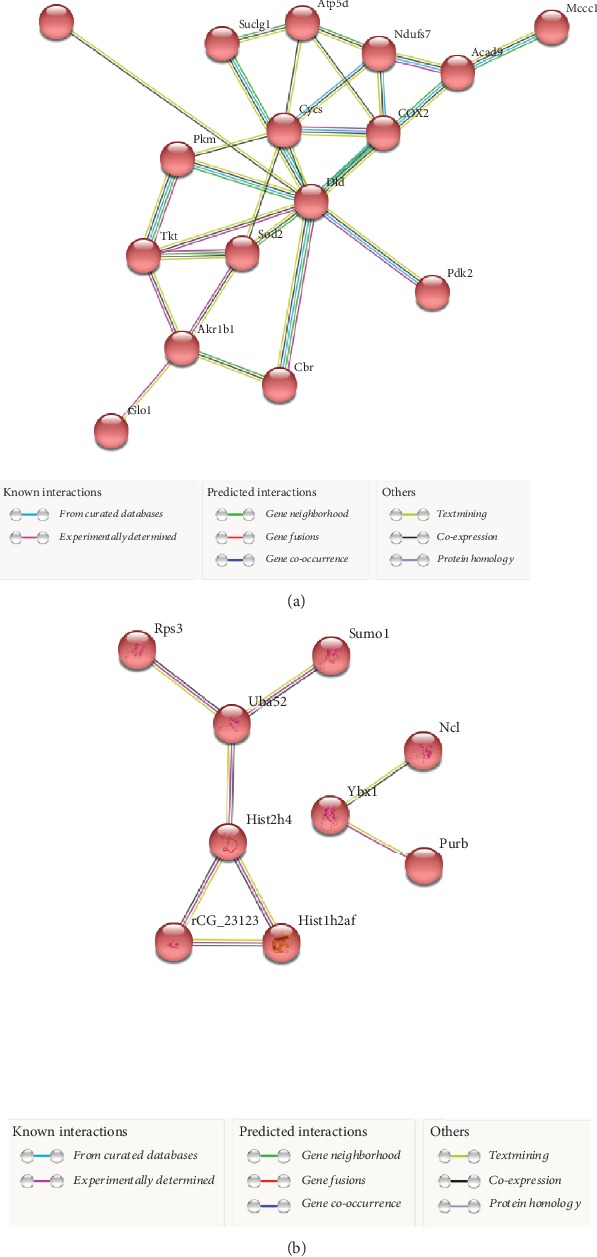
Protein-protein interaction analysis based on STRING with an interaction confidence score (0.400). SFO-HF: offspring from sedentary fathers exposed to high-fat diet; TFO-HF: offspring from trained fathers exposed to high-fat diet. STRING analysis for differentially abundant proteins upregulated in the analysis (TFO-HF : SFO-HF). Red highlighted nodes are related to metabolic processes including oxidative stress protection, glycolysis pathway, mitochondrial respiratory chain, and fatty acid metabolism proteins (a) and to translation, nucleosome assembly, and transcription regulation (b).

STRING analysis revealed protein networks related to metabolic processes in TFO-HF : SFO-HF, showing high connectivity among oxidative stress protection, glycolysis, mitochondrial respiratory chain, and fatty acid metabolism proteins (Figure 7(a)). A prominent interaction network was found within proteins, such as methionine sulfoxide reductase B3, dihydrolipoyl dehydrogenase, glutathione S-transferase Mu 2, isoform M2 of pyruvate kinase PKM, and acyl-CoA dehydrogenase family member 9. We also found that upregulated proteins related to translation, nucleosome assembly, and transcription regulation interacted with each other, including histone H2A, histone H2B, histone H4, nucleolin, and 40S ribosomal protein S3 (Figure 7(b)). No significant protein networks were observed between the other groups and biological processes.

## 4. Discussion

Preconceptional paternal RT upregulates protein abundance levels related to myofibril components, cell processes, metabolic processes, antioxidant activity, transport, nucleosome assembly, translation, and transcription regulation on the LV regardless of the offspring diet, which may be crucial for heart homeostasis and cellular function. In this context, overlapping proteins, which change in the same direction (i.e., upregulation or downregulation), were present in all of the three groups involved in RT (i.e., TF, TFO-C, and TFO-HF groups).

To our knowledge, decreased protein abundance of phospholemman and upregulation of spectrin beta chain in the LV after RT are described for the first time in the present study. In the heart, phospholemman represents a direct molecular link between the regulation of Na^+^/K^+^ pump and Na^+^/Ca2^+^ exchanger activities in response to cellular signaling cascades mediated by protein kinases [31]. When the heart is under stress, phospholemman enhances Na^+^K^+^-ATPase activity minimizing risks of arrhythmogenesis, thereby at the expense of reduced inotropy [32]. Decreased phospholemman reduces Na^+^K^+^ pump activity, promoting contractile dysfunction [33]. The downregulation of this protein might represent an adaptation mechanism induced by RT to prevent inotropism reduction, likely as a compensatory process. Regarding the spectrin beta chain, it has been reported as a crucial component in maintaining cardiac membrane excitability [34]. Spectrin beta chain downregulation is associated with heart failure and maladaptive cardiac remodeling [35]. However, this protein was upregulated by RT, which indicates regulatory effects on electrical functions in cardiomyocytes.

The RT provided significant structural and contractile protein upregulation in fathers. The keratin overexpression can indicate a compensatory mechanism that maintained intercalated discs and epithelial tissue homeostasis [36]. The RT led to the downregulation of respiratory electron transport chain proteins. Dantas et al. [18] found an overexpression of respiratory chain proteins on the LV of rats after RT. However, used weighted aquatic jumps possibly have a more considerable aerobic component when compared to weighted stair climbing. This current result is similar to Schoepe et al. [37] that demonstrated decreased activity on respiratory chain complexes I and IV in the heart after 6 weeks of aerobic training. The authors demonstrated that cardiac oxidative capacity was further increased after 10 weeks of training, suggesting that this activity reduction is temporary. We might speculate that aerobic exercise can increase respiratory chain proteins in the LV, while the RT beneficial effects would have a later onset.

In the current study, both offspring from trained fathers showed a reduction in the abundance of three proteins related to respiratory electron transport chain (ATP synthase complex subunit B1, mitochondrial; ATP synthase subunit O, mitochondrial; and cytochrome b-c1 complex subunit 1) when compared with offspring from sedentary fathers, which suggests that paternal training modality may be crucial for a better understanding of the offspring adaptations. The lack of difference in distance and aerobic capacity in the incremental-speed treadmill running test might be due to the downregulation of these proteins. Future studies may wish to compare the effects of different exercise modalities (resistance, aerobic, and combined) on molecular mechanisms involved in transgenerational inheritance.

An important finding was that SFO-HF demonstrated a decrease on proteins related to muscle contraction, immune response, and transport when compared with the SFO-C group. The cardiac phospholamban protein plays an important role in cardiac contractility through calcium cycling and is a crucial determinant of *β*-adrenergic stimulation [38]. Reduction of this protein might be related to myocardial dysfunction, increasing heart failure risk in the SFO-HF group. In addition, decreased complement component 4A and parathymosin proteins suggest a reduction in immune system effectiveness, thus predisposing the animal to opportunistic infections [39, 40]. Finally, hypovitaminosis D plays an important role in the development of heart failure, myocardial infarction, and arterial hypertension [41]. The decreased vitamin D-binding protein may indicate a transport deficit of vitamin D metabolites, leading to an increase of fatty acid polymerization, which could be detrimental in the circulatory system [42].

The TFO-HF group displayed higher protein abundance levels related to muscle contraction when compared to the TFO-C group. These findings suggest that the association between paternal resistance training and HF diet induces more significant LV structural adaptations than isolated HF diet. Although offspring exposure to HF diet may result in pathologic cardiac hypertrophy [43], paternal RT possibly normalizes heart plasticity. Concomitantly, the proteins related to metabolic processes can be associated with elevated energy demands in cardiomyocyte due to paternal training and HF diet exposure. Otherwise, S100-A6 protein, junctophilin, and cardiac phospholamban protein abundance levels were shown to be reduced in the TFO-HF group, since these proteins play an import role in calcium homeostasis maintenance [44, 45].

It is worthy to point out that essential antioxidant proteins such as glutathione, methionine sulfoxide reductase, peroxiredoxin, ferritin, and albumin were upregulated in the three groups involved in exercise training (i.e., TF, TFO-C, and TFO-HF groups) when compared with sedentary groups, which undergird the reliability and increase the importance of the findings. In other words, increased antioxidant defense induced by long-term paternal training can preserve cardiac mitochondria redox status, which is an important adaption in the first-line defense mechanisms against oxidative stress. Cellular reduction-oxidation balance plays a crucial role in heart function recovery and homeostasis. Reactive oxygen species (ROS) may contribute to the initiation and development of the pathological process, while an increase of antioxidant proteins can be important to maintain enzymatic operation in the heart metabolism, possibly preventing impairments in mitochondrial oxidative capacity [46].

The overproduction of ROS induced by HF diet, along with oxidant/antioxidant imbalance, can lead to oxidative stress and related tissue damage [46]. Previous studies have shown that RT is capable of upregulating nuclear factor erythroid-2-related factor 2 protein, the primary regulator of endogenous antioxidant defenses [47, 48]. In this context, paternal RT can promote offspring cardioprotection via a redox-based mechanism against possible deleterious action of ROS in extreme stress situations. This fact would help to prevent apoptosis, damage to cellular structures, and cardiac injury. Considering these molecular findings, we can state that paternal RT can be used to produce greater key protein regulation for cellular survival and energy metabolite homeostasis, which can minimize the deleterious effects of the HF diet and avoid major damages on the LV. We hypothesize that these adaptations probably occur to prevent the cardiac dysfunction onset as an attempt to reinvigorate the heart. Relevantly, the TFO-HF group possibly has less vulnerable cardiomyocytes to cardiac insults and more capable of coping with oxidative stress due to an increase of several antioxidant and transport proteins involved in this protection process.

Paternal RT was also effective in preventing body and adipose tissue weight gain in the offspring exposed to HF diet. This result contrasts with Murashov et al. [49] that investigated the effects of voluntary wheel running in C57BL/6J mice on their offspring's predisposition to insulin resistance and body weight. The authors showed that fathers subjected to wheel running produced offspring that were susceptible to the unfavorable outcomes of HF diet, displayed increased adiposity, and impaired glucose tolerance. There are methodological explanations for discrepancy between the studies: different species, diet time exposure, and training variables, which may attenuate beneficial effects. Another novelty was that paternal RT attenuated LV weight gain, which indicates that paternal RT can promote delay in pathologic hypertrophy.

Paternal RT did not result in decreased glucose AUC. Krout et al. [11] showed that paternal exercise was protective against insulin resistance by increasing the expression of insulin signaling markers in skeletal muscle resulting in normal T2D risk in offspring. The difference between the present study and Krout et al. [11] is the utilization of animals from different species. The C57BL/6J lineage mice respond more pronouncedly to HF diet, which can facilitate insulin resistance when compared to Wistar rats [50]. Also, a large amount of protein in HF diet might delay the onset of insulin insensitivity [51]; however, the authors did not present the protein composition of the diet. Regarding the plasma lipid profile, paternal RT was not able to prevent any parameters. The effects of HF diet on lipid profile are controversial due to variations in the HF diet administered; moreover, the lipid profile is associated with the increase in unsaturated fatty acid and carbohydrate consumption and not only to HF diet [52].

The voltage-dependent R-type calcium channel (CACNA) increased in TFO-HF when compared with SFO-HF, which suggests that paternal RT can be potentially important for maintaining normal contractility of the offspring heart. Molina-Navarro et al. [53] showed the downregulation of CACNA in the patients with dilated cardiomyopathy when compared to healthy individuals, which suggests that a decrease of this channel may be related with pathological condition.

The TFO-HF : SFO-HF analysis detected more regulated proteins related to metabolic processes than any other comparison or biological process. In the normal heart, fatty acid oxidation accounts for 60% of myocardium energy demand, with the remaining provided by glucose and pyruvate metabolism [54]. The increased fatty acid uptake and concomitantly decreased glucose metabolism result in larger total production of ATP, but a reduction in the amount of ATP produced per mole of oxygen consumed [55]. In the heart failure, the metabolic switch towards favoring glucose oxidation over fatty acid oxidation had been considered a maladaptive change [56]. However, glucose dependence is not, apparently, harmful in adult hearts and decreased glucose utilization seems to be deleterious in heart failure [56]. Our findings suggest that paternal RT might improve offspring cardiomyocyte energy efficiency.

Essential proteins related to transporting molecules involved in metabolism and ions bioavailability were increased in the TFO-HF group. The myoglobin regulates mitochondrial O_2_ supply and diffusion, contributing to the protection of the respiratory chain, moreover balancing the nitric oxide level in the cardiomyocytes [57]. Ferritins contribute to maintenance of iron concentrates for cofactor syntheses and sequestration of iron from invading pathogens [58]. Solute carrier family 25 member 12 is important for calcium-binding mitochondrial carrier protein and reduced glucose-induced oxidative metabolism [59]. These molecular findings suggest that paternal RT can be relevant for cellular survival and energy metabolite homeostasis.

**Figure 8:**
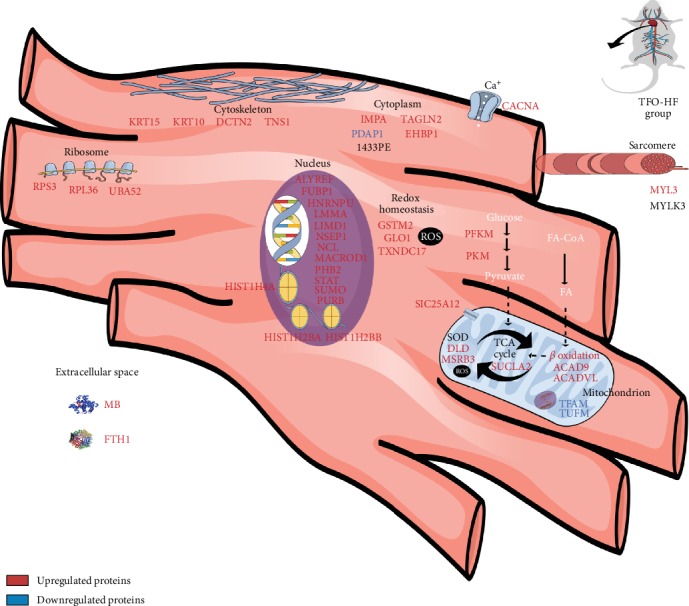
Modulation in the cardiomyocyte proteome. SFO-HF: offspring from sedentary fathers exposed to high-fat diet; TFO-HF: offspring from trained fathers exposed to high-fat diet. The main proteins upregulated (red) and downregulated (blue) in the analysis (TFO-HF : SFO-HF). Cell membrane: voltage-dependent R-type calcium channel subunit alpha (CACNA). Cytoplasm: 28 kDa heat- and acid-stable phosphoprotein (PDAP1), ATP-dependent 6-phosphofructokinase (PFKM), EH domain-binding protein 1-like 1 (EHBP1), and pyruvate kinase (PKM). Cytoskeleton: keratin, type I cytoskeletal 10 (KRT10); keratin, type I cytoskeletal 15 (KRT15); and tensin 1 (TNS1). Extracellular space: ferritin (FTH1) and myoglobin (MB). Isoform 2 of tyrosine-protein phosphatase nonreceptor type 11 (PTN11). Mitochondrion: acyl-CoA dehydrogenase family member 9 (ACAD9); acyl-CoA dehydrogenase, very long chain (ACADVL); ATP synthase F(0) complex subunit B1 (AT5F1); ATP synthase subunit O (ATPO); cytochrome b-c1 complex subunit 1, mitochondrial (QCR1); methionine sulfoxide reductase B3 (MSRB3); succinate-CoA ligase (SUCLA2); and transcription factor A, mitochondrial (TFAM). Nucleus: Aly/REF export factor (ALYREF), far upstream element-binding protein 1 (FUBP1), heterogeneous nuclear ribonucleoprotein U (HNRNPU), histone H2A (HIST1H2BA), histone H2B (HIST1H2BB), histone H4 (HIST1H4A), lamin A, isoform CRA_b (LMMA), LIM domain-containing protein 1 (LIMD1), nuclease-sensitive element-binding protein 1 (NSEP1), nucleolin (NCL), O acetyl-ADP-ribose deacetylase MACROD1 (MACROD1), prohibitin-2 (PHB2), signal transducer and activator of transcription (STAT), small ubiquitin-related modifier (SUMO), and transcriptional activator protein Pur-beta (PURB). Redox homeostasis: glutathione S-transferase Mu 2 (GSTM2), lactoylglutathione lyase (GLO1), and thioredoxin domain-containing protein 17 (TXNDC17). Ribosome: 40S ribosomal protein S3 (RPS3), ribosomal protein L36-like (RPL36), and ubiquitin-60S ribosomal protein L40 (UBA52). Sarcomere: isoform 3 of myosin light chain (MYL3).

The offspring from trained fathers increased the histone proteins when compared with offspring from sedentary fathers. Paternal RT might be linked to a physiological status more prone to programming transcription [60]. Our findings demonstrated higher protein abundance levels associated with nucleosome assembly, translation, and transcription regulation on the TFO-HF group. An illustration of cardiomyocyte was used to clarify the location and the upregulation and downregulation of main proteins in the TFO-HF : SFO-HF analysis (Figure 8).

Some limitations of the present study should be highlighted, such as the impossibility to analyze immunoblot analysis of key proteins, gene expression, morphology properties of the LV, and ventricular functional assessments. In addition, future studies are aimed at defining sperm epigenetic status of fathers. Lastly, microRNAs are epigenetic mechanisms capable of influencing offspring phenotype [10, 49].

## 5. Conclusions

It was identified that the father's lifestyle and offspring diet significantly modified the LV proteome, distinctly altering protein abundance levels. The offspring HF diet led to decreased protein abundance levels related to cell component organization, immune response, transport, and translation, which may potentiate LV function worsening. On the other hand, the beneficial effects of paternal RT on the LV proteome are independent of offspring diet. Proteomic analysis demonstrated that paternal RT is a critical factor capable of reprogramming offspring LV proteins associated with muscle contraction, metabolic processes, antioxidant activity, transport, and transcription regulation. The present research provides valuable insights into the molecular mechanisms involved in paternal transgenerational inheritance.

## Figures and Tables

**Table 1:** Protein abundance levels related to miscellaneous functions from TFO-HF : TFO-C and TFO-HF : SFO-HF analysis considering only proteins with upregulation and downregulation (*p* ≤ 0.05), with a delta (Δ) of at least (≥) 0.5-fold change.

Protein description	Primary name	Biological process	*p* value	Log(*e*) fold change
TFO-HF : SFO-HF				
28 kDa heat- and acid-stable phosphoprotein	HAP28_RAT	Cell proliferation	0.0001	0.642709314
Annexin	ANXA1_RAT	Blood coagulation	0.0001	0.620131631
cAMP-dependent protein kinase inhibitor alpha	P63249|IPKA_RAT	Biological regulation	0.0001	0.551039992
Dynactin subunit 2	DCTN2_RAT	Cell cycle	<0.0001	0.518489876
Inter-alpha-trypsin inhibitor, heavy chain 4	ITIH4_RAT	Inflammatory response	0.0003	0.782536172
Isoform 2 of G-protein-signaling modulator 1	Q9R080-2|GPSM1_RAT	Neurogenesis	0.0235	0.602697354
Kininogen-1	P08934|KNG1_RAT	Blood coagulation	0.0005	0.657253874
NSFL1 cofactor p47	NSF1C_RAT	Biological regulation	<0.0001	0.637752488
Protein disulfide-isomerase A6	PDIA6_RAT	Protein folding	0.0165	0.58285564
Protein-L-isoaspartate(D-aspartate) O-methyltransferase	PIMT_RAT	Protein repair	<0.0001	1.015385531
Protein phosphatase 2, regulatory subunit A	Q5XI34_RAT	Cell cycle	0.0145	1.125946751
Retinoid-inducible serine carboxypeptidase	RISC_RAT	Biological regulation	0.0212	0.691632518
Serine proteinase inhibitor, clade H, member 1, isoform CRA_b	Q5RJR9_RAT	Biosynthetic process	0.0016	0.894924934
Serine/threonine-protein kinase mTOR	MTOR_RAT	Biological regulation	0.0027	0.774556009
Tensin 1	F1LN42_RAT	Cell adhesion	0.0157	0.517836179
TFO-HF : TFO-C				
Beta-2-microglobulin	B2MG_RAT	Immune response	0.0028	-0.957888096
Bifunctional purine biosynthesis protein PURH	PUR9_RAT	Biosynthetic process	<0.0001	0.678632547
Cardiac phospholamban	PPLA_RAT	Biological regulation	<0.0001	-0.955260487
Caveolae-associated protein 3	CAVN3_RAT	Biological rhythms	0.0002	-1.375582354
Eukaryotic translation initiation factor 4B	Q5RKG9|Q5RKG9_RAT	Biosynthetic process	0.011	-0.74609642
Fibrinogen beta chain	FIBB_RAT	Blood coagulation	0.0043	0.705823625
Guanylate-binding protein 1	GBP1_RAT	Immune response	0.0291	1.253583879
Inter-alpha-trypsin inhibitor, heavy chain 4	ITIH4_RAT	Inflammatory response	0.0007	0.620646553
Isoform 2 of tyrosine-protein phosphatase nonreceptor type 11	PTN11_RAT	Biological regulation	<0.0001	-0.612738686
Protein disulfide-isomerase A6	PDIA6_RAT	Protein folding	0.0137	0.617987716
Protein S100-A6	S10A6_RAT	Cell cycle	0.0025	-1.150239715
Reticulon 4 interacting protein 1	RT4I1_RAT	Neurogenesis	0.0114	0.747496638
Serine proteinase inhibitor, clade H, member 1, isoform CRA_b	Q5RJR9_RAT	Biosynthetic process	0.0009	1.181489284
Serine/threonine-protein kinase mTOR	MTOR_RAT	Biological regulation	0.0019	0.87608699

**Table 2:** *p* for interaction values between paternal training and offspring diet in protein abundance levels controlling body and tissue weights.

Protein description	Primary name	Biological process	*p* value Interaction
Acad9 protein	B1WC61_RAT	Metabolic process	0.02
Amine oxidase	G3V9Z3_RAT	Metabolic process	0.01
Annexin	ANXA1_RAT	Blood coagulation	0.01
Aly/REF export factor	D3ZXH7_RAT	Transcription	0.02
ATP synthase subunit beta	G3V6D3_RAT	Metabolic process	0.03
ATP synthase subunit delta, mitochondrial	ATPD_RAT	Metabolic process	0.0001
Beta-enolase	ENOB_RAT	Metabolic process	0.003
Carbonyl reductase (NADPH) 1	CBR1_RAT	Metabolic process	0.0005
Cardiac phospholamban	PPLA_RAT	Biological regulation	0.001
CArG-binding factor A	Q9QX80_RAT	Biological regulation	0.002
Caveolae-associated protein 3	CAVN3_RAT	Biological rhythms	0.01
Cytochrome b-c1 complex subunit 2, mitochondrial	QCR2_RAT	Respiratory electron transport	0.001
Dynamin-1-like protein	DNM1L_RAT	Cell component organization	0.01
Dystrobrevin	D4A772_RAT	Muscle contraction	0.03
EH domain-binding protein 1-like 1	A0A0G2K6R8_RAT	Transport	0.0003
Glutathione S-transferase Mu 2	GSTM2_RAT	Metabolic process	0.006
Guanylate-binding protein 1	GBP1_RAT	Immune response	0.005
Heterogeneous nuclear ribonucleoprotein A1	ROA1_RAT	Transport	0.01
Histone H2A	D4ACV3_RAT	Nucleosome assembly	0.02
Histone H2B	H2B1_RAT	Nucleosome assembly	0.02
Inter-alpha-trypsin inhibitor, heavy chain 4	ITIH4_RAT	Inflammatory response	0.003
Isoform 2 of G-protein-signaling modulator 1	GPSM1_RAT	Neurogenesis	0.04
Isoform 2 of tyrosine-protein phosphatase nonreceptor type 11	PTN11_RAT	Biological regulation	0.0005
Isoform short of mitochondrial pyruvate carrier 2	MPC2_RAT	Transport	0.04
Lactoylglutathione lyase	LGUL_RAT	Metabolic process	0.007
Lamin A, isoform CRA_b	G3V8L3_RAT	Chromatin organization	0.001
Methionine sulfoxide reductase B3	MSRB3_RAT	Oxidation-reduction process	0.003
Myoglobin	MYG_RAT	Transport	0.01
NSFL1 cofactor p47	NSF1C_RAT	Biological regulation	0.05
Phosphate carrier protein, mitochondrial	MPCP_RAT	Transport	0.03
Protein disulfide-isomerase A6	PDIA6_RAT	Protein folding	0.03
Protein-L-isoaspartate(D-aspartate) O-methyltransferase	PIMT_RAT	Protein repair	0.0002
Protein phosphatase 2 (formerly 2A), regulatory subunit A (PR 65), alpha isoform,	Q5XI34_RAT	Cell cycle	0.001
Pyruvate dehydrogenase (acetyl-transferring) kinase isozyme 2, mitochondrial	PDK2_RAT	Metabolic process	0.04
RCSD domain containing 1 (predicted), isoform CRA_a	A0A0G2K7I4_RAT	Muscle contraction	0.01
Ribosomal protein L10-like	RL10L_RAT	Translation	0.02
Ribosomal protein L36-like	RL36_RAT	Translation	0.01
Serine (or cysteine) proteinase inhibitor, clade H, member 1, isoform CRA_b	Q5RJR9_RAT	Biosynthetic process	0.005
Serine/threonine-protein kinase mTOR	MTOR_RAT	Biological regulation	0.006
Succinate-CoA ligase (GDP-forming) subunit beta, mitochondrial	SUCB2_RAT	Metabolic process	0.001
Tescalcin	D3ZTN1_RAT	Transport	0.01
Transcription factor A, mitochondrial	TFAM_RAT	Transcription regulation	0.009
Tropomyosin alpha-1 chain	TPM1_RAT	Muscle contraction	0.05

## Data Availability

Supplementary data 2 used to support the findings of this study are included within the article.

## Supplementary Materials

Supplementary 1 Supplementary data 1: composition of the experimental diets. Supplementary figure 1: classification of the identified proteins according to their cellular localization (A), biologic process (B), and molecular function (C). Supplementary figure 2: Venn diagram representation of the identified proteins by Nano-UPLC-MS analysis in the sedentary father (SF) and trained father (TF) groups. Proteins exclusive to TF and SF were classified by their biological process. Supplementary figure 3: Venn diagram representation of the identified proteins by Nano-UPLC-MS analysis in the four offspring groups (SFO-C, TFO-C, SFO-HF, and TFO-HF). The identified proteins exclusive to the TFO-HF group were classified by their biological process.

Supplementary 2 Supplementary data 2: normalized abundance.

Supplementary 3 Supplementary data 3: two-way ANCOVA analyses.
