# Supplementary material for: Paternal Resistance Training Induced Modifications in the Left Ventricle Proteome Independent of Offspring Diet
Source: Oxid Med Cell Longev. 2020 May 4;2020:5603580. doi: 10.1155/2020/5603580 (PMC7218999; doi:10.1155/2020/5603580)

Supplementary data 1

Composition of the experimental diets

Nutrients	Diets	
	Standard chow g%	High-fat diet g%
Proteins	24	19.89
Lipids	10	59.38
Carbohydrates	66	20.27
Fiber (Celulose)	5.0	6.46
Mineral mix	3.7	12.9
Vitamin mix	1.9	1.29
Choline bitartrate	0.2	2.60
Lard	0.0	3.16
Soy oil	0.0	3.23
Total energy (kCal/g)	3.48	5.2

Source: The calculations of chemical composition were based on nutritional information sent by the supplier of the products

Supplementary figure 1: Classification of the identified proteins according to their cellular localization (A), biologic process (B), and molecular function (C).

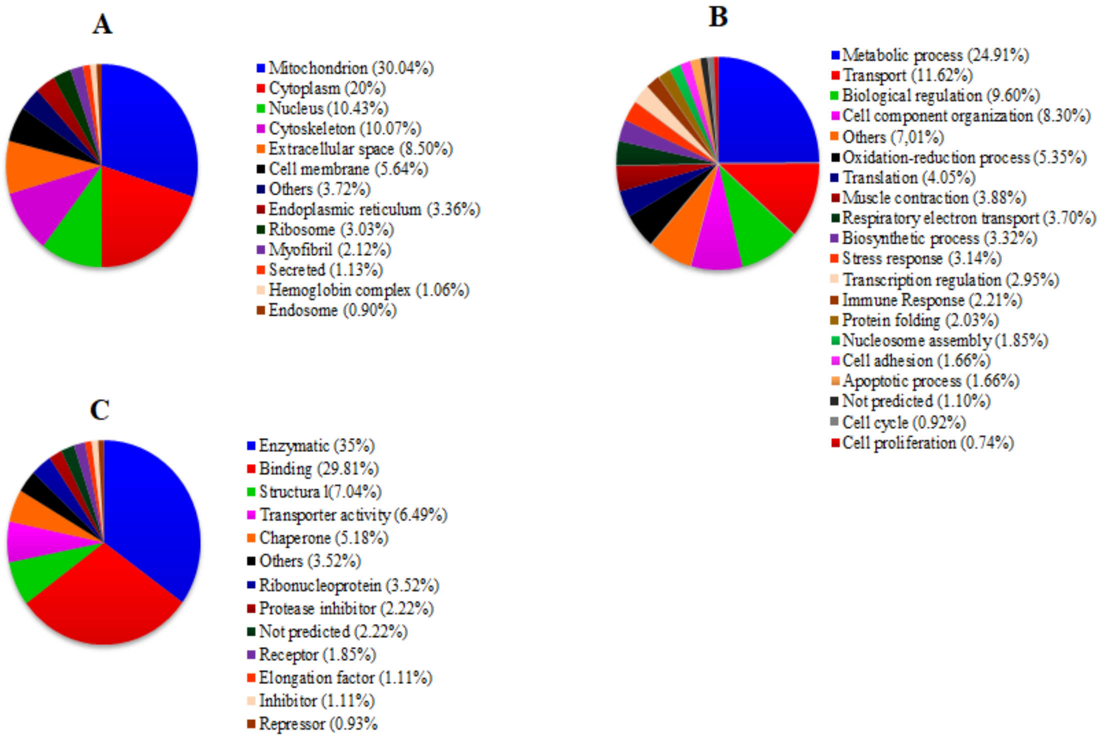

Supplementary figure 2 Venn diagram representation of the identified proteins by NanoUPLC-MS analysis in the Sedentary Fathers (SF) and Trained Fathers (TF) group. Proteins exclusive to TF and SF were classified by their biological process.

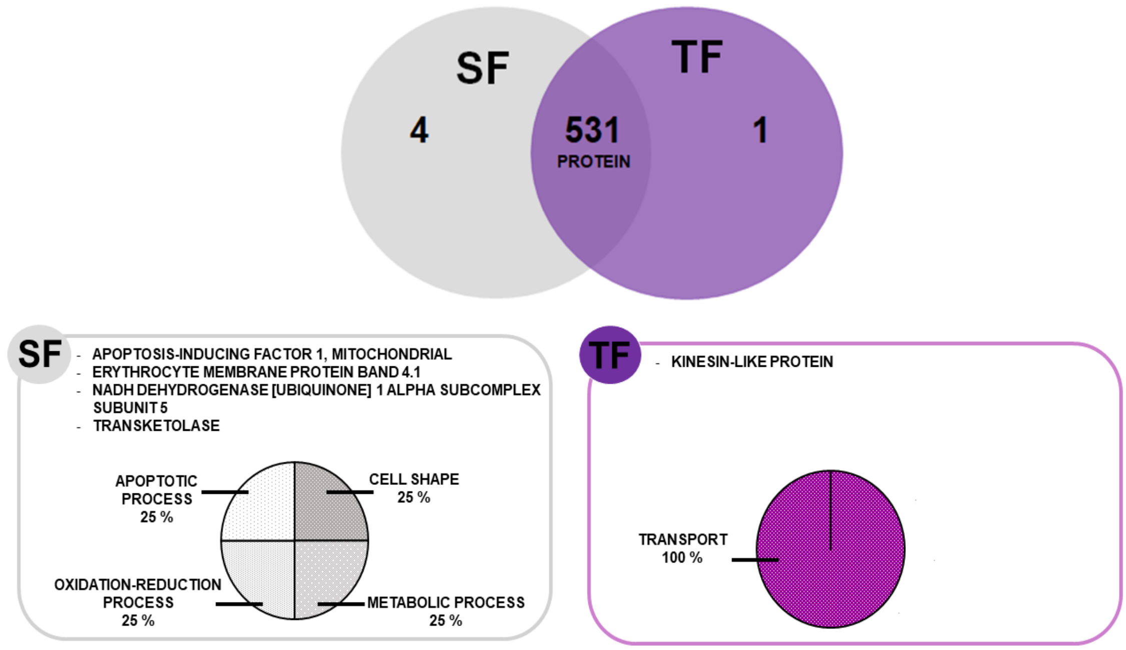

Supplementary figure 3 Venn diagram representation of the identified proteins by NanoUPLC-MS analysis in the four offspring groups (SFO-C; TFO-C; SFO-HF; TFO-HF). The identified protein exclusive to the TFO-HF group were classified by their biological process.

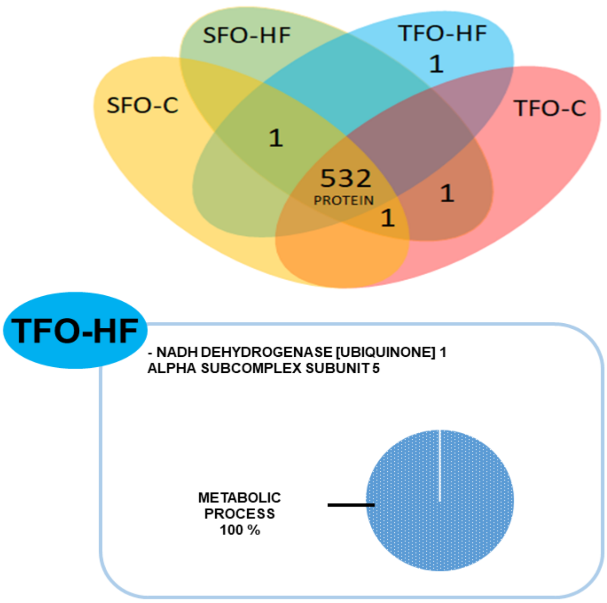

Supplement: Supplementary 1 — Supplementary data 1: composition of the experimental diets. Supplementary figure 1: classification of the identified proteins according to their cellular localization (A), biologic process (B), and molecular function (C). Supplementary figure 2: Venn diagram representation of the identified proteins by Nano-UPLC-MS analysis in the sedentary father (SF) and trained father (TF) groups. Proteins exclusive to TF and SF were classified by their biological process. Supplementary figure 3: Venn diagram representation of the identified proteins by Nano-UPLC-MS analysis in the four offspring groups (SFO-C, TFO-C, SFO-HF, and TFO-HF). The identified proteins exclusive to the TFO-HF group were classified by their biological process. [file 5603580.f1.pdf]
